# Shennongjia–Wushan Mountains—One cryptic glacial refugium introduced by the phylogeographical study of the Geometridae moth *Ourapteryx szechuana* Wehrli

**DOI:** 10.1002/ece3.7794

**Published:** 2021-06-21

**Authors:** Rui Cheng, Hongxiang Han, Dayong Xue, Chaodong Zhu, Nan Jiang

**Affiliations:** ^1^ Key Laboratory of Zoological Systematics and Evolution Institute of Zoology Chinese Academy of Sciences Beijing China

## Abstract

The origin and evolution of biodiversity in the Shennongjia and Wushan Mountains, located in central China, are little known. In this study, we used *Ourapteryx szechuana*, which is widely distributed in China and northern Nepal, to explore whether these mountains acted as glacial refugia during climate oscillations of the Quaternary. In total, 192 samples of *O. szechuana* were collected throughout much of the distribution range. Phylogenetic analysis, molecular dating, demographic history reconstructions, and MAXENT were used to investigate the evolutionary history and differentiation mechanisms and predict the potential species distributions during four different periods. The phylogenetic tree and the star‐like median‐joining network strongly supported two reciprocally monophyletic and allopatric lineages. Lineage I was restricted to the Shennongjia and Wushan Mountains. The divergence time of *O. szechuana* from its sister species *O. thibetaria* was approximately 1.94 Ma. The differentiation processes of the two intraspecific lineages occurred at approximately 0.47 Ma. The demographic history reconstruction and the ecological niche model suggested that Lineage II experienced an expansion after the LGM (Last Glacial Maximum), whereas Lineage I did not experience any expansion. Our results suggested the Naynayxungla glaciation promoted the divergence of the two lineages by restricting them to different refugia. The valleys of the Shennongjia–Wushan Mountains may have kept stable and warm (thus ice‐free) environments during Quaternary glaciations, allowing this region to act as a glacial refugia. Our studies show that the Shennongjia and Wushan Mountains are likely to be important but little studied glacial refugia for the insect and thus worthy of more attention.

## Graphical abstract

The Naynayxungla Glaciation promoted the divergence of the two lineages by restricting them to different refugia. The Shennongjia and Wushan Mountains are one important and easily neglected refugia for the insect groups and are worthy of more attention.

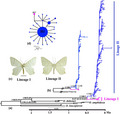

## 1 INTRODUCTION

During the Quaternary Period, climatic fluctuations during glacial–interglacial cycles may be the most dominant paleoclimatic events, which have greatly influenced species distribution, genetic diversification, and demography (Hewitt, 1996, 2000). Studies of European and North American taxa found that species were generally restricted to southern refugia during the Last Glacial Maximum (LGM, 0.021–0.018 million years ago, Ma) and expanded northwards as the climate warmed up. Compared with detailed research conducted in Europe, relatively few investigations have been focusing on other parts of the world, such as East Asia (Beheregaray, 2008), although this region is an important biogeographical area with high species diversity (Myers et al., 2000; Qian & Ricklefs, 2000) and many endemic species (Lei et al., 2003). Previous studies revealed that the effects of climatic fluctuations were milder and more complicated in East Asia than in Europe and North America and are not restricted to the LGM (Cheng, Jiang, Yang, et al., 2016; Wu et al., 2002). Three other main glaciations (Xixiabangma, Naynayxungla, and Guxiang glaciations) (Zheng et al., 2002) also had important influences on East Asian species (Cheng, Jiang, Yang, et al., 2016; Cheng et al., 2017; Li et al., 2017; Qiu et al., 2009; Qu et al., 2012).

The term “refugium” is commonly used in biology to designate the sites where a wide array of both animal and plant species lived during glacial periods throughout the Quaternary (Hewitt, 1996, 2000; López‐Pujol et al., 2011). During ice ages, when the climate was significantly colder and drier, the distribution of species changed significantly, which resulted in range contractions and/or species displacement toward sites with suitable ecological conditions (Davis & Shaw, 2001; Hu et al., 2009). During cyclical phases of climate‐induced allopatry, some extent of genetic differentiation can sometimes lead to the development of new species/subspecies, which is congruent with the “glacial refugium hypothesis” (Holder et al., 1999; Mengel, 1964; Stewart et al., 2009; Wielstra et al., 2020). Previous studies have proven that the Quaternary refugia are often located in mountainous areas because of their dual role of “evolutionary museums and cradles” (López‐Pujol et al., 2011; Tzedakis et al., 2002). Thus, China, as a mountainous country, retains multiple refugia (Myers et al., 2000), for example, the Hengduan Mountains, the Qinling‐Daba Mountains, and the Nanling Mountains, all of which have been supported as refugia in multiple taxa, such as plants (Qiu et al., 2009; Ren et al., 2020; Tian et al., 2009), birds (Qu et al., 2012; Wang et al., 2013; Zou et al., 2007), and insects (Cheng, Jiang, Xue, et al., 2016; Li et al., 2017; Morgan et al., 2011). Compared with these well‐known refugia, some other regions located in central China (such as the Shennongjia and Wushan mountains) host relatively less species, but may nevertheless have played a significant, yet underestimated role. So far, this role has only been investigated in plant research (Lei et al., 2012; Yao et al., 2012).

Accurately identifying glacial refugia is a high priority for conservation because they are key areas for investigating the persistence and evolution of biodiversity (Lau et al., 2020; López‐Pujol et al., 2011; Shen et al., 2002; Tzedakis et al., 2002). In the beginning, the identification of refugia was mainly based on the distribution of endemic species, fossils, and pollen (Hewitt, 2000; Petit et al., 2002; Willis, 1996). Fortunately, molecular phylogeographic studies have provided independent data for identifying possible refugia and tracking the colonization routes for many animals and plants (Lei et al., 2012; Liu et al., 2016; Wang et al., 2013). At present, numerous studies have outlined the phylogeography of European and North American fauna (Hewitt, 1996, 2000; Lefebvre et al., 2016; McDevitt et al., 2020). Despite a remarkable biodiversity and a very wide range of climatic conditions, only a relatively moderate number of studies have tackled the phylogeography of Chinese organisms, and evidence is lacking particularly in insects (Cheng et al., 2019; Cheng, Jiang, Yang, et al., 2016; Li et al., 2017). Previous studies have suggested a general pattern of multiple refugia with little admixture among refugial populations (Hewitt, 1996, 2000; Wang & Ge, 2006; Lei et al., 2012). However, it would be of great interest to know whether this pattern is also applicable to more widespread and common species in China.

*Ourapteryx szechuana* Wehrli, 1939, (Geometridae, Ennominae) is widely distributed in most regions of China and northern Nepal (Cheng et al., 2019). It is common in mountain areas and found at altitudes from 213 to 3,340 m. Here, we used mitochondrial DNA (mtDNA) and nuclear DNA (ncDNA) to examine the evolutionary history of *O. szechuana*. Specifically, we (i) investigated the phylogeographical patterns and the divergence times of *O. szechuana* on the basis of molecular evidence, (ii) tested the influence of topography and climatic fluctuations on lineages, (iii) explored possible refugia in China based on phylogeographical methods, and (iv) studied the occurrence of *Wolbachia* infection to test whether it varied throughout the distribution range of two lineages of *O. szechuana*.

## 2 MATERIALS AND METHODS

### 2.1 Sampling and sequence data

**FIGURE 1:**
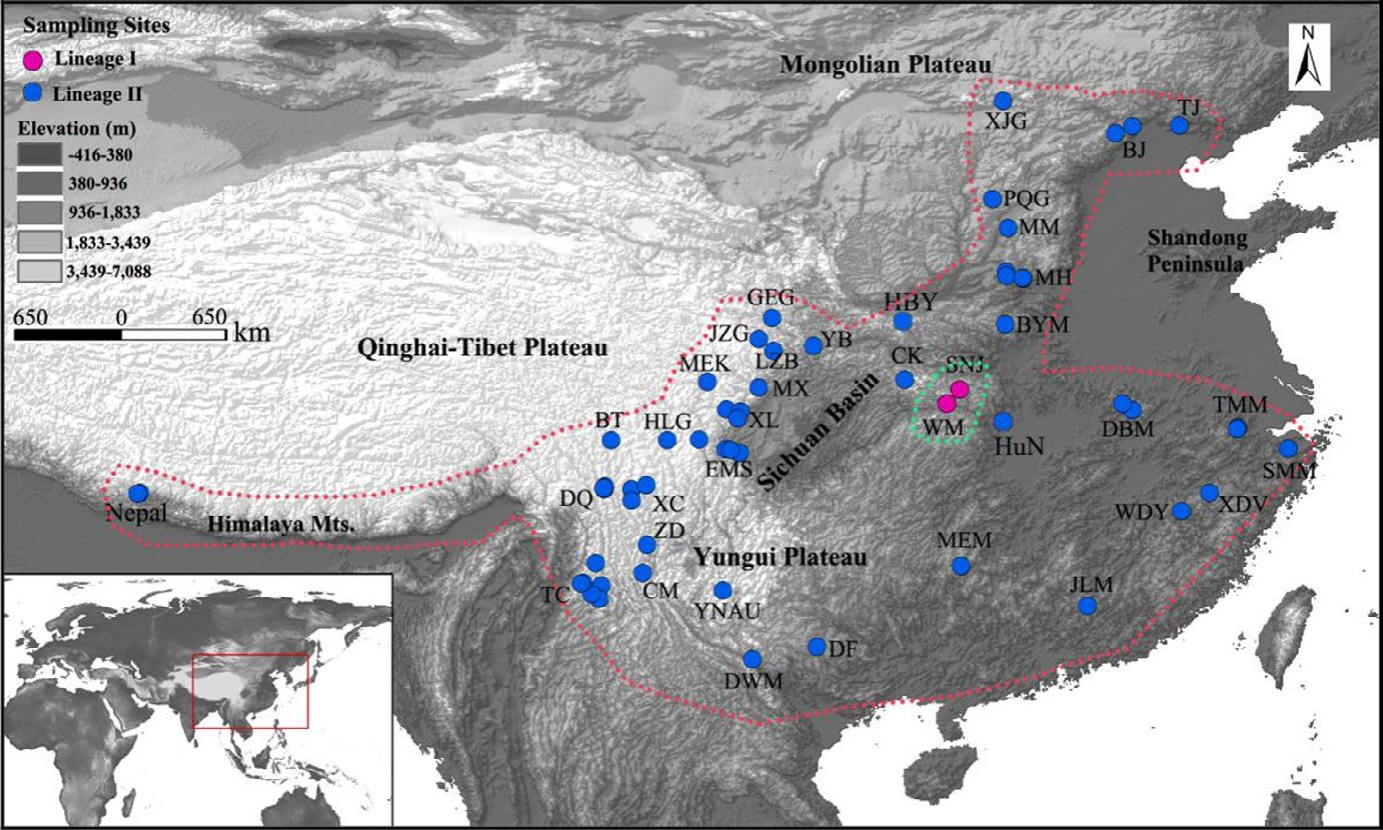
Sampling sites of *O. szechuana* used in this study. Colors represent different lineages

A total of 192 specimens of *O. szechuana* were collected from 64 sampling sites throughout much of the species’ distribution range. We pooled the 64 sampling sites into 42 geographical locations (for the abbreviations of locations, see Figure 1 and Table S1 in the Supporting Information). The samples for DNA extraction were preserved in 100% ethanol and stored at −20℃. DNA was extracted using the DNeasy Tissue Kit (Qiagen, Beijing, China), and the vouchers were deposited at the Museum of IZCAS (Institute of Zoology, Chinese Academy of Sciences, Beijing, China). Four mitochondrial and three nuclear genes were obtained, including COI, CYTB, COII, ND5, EF‐1a, GAPDH, and CAD genes, through polymerase chain reaction (PCR) amplification. The four mtDNA loci were amplified as described in (Cheng, Jiang, Xue, et al., 2016, Cheng, Jiang, et al., 2019). The three ncDNA loci were amplified as described in Ban et al. (2018). The sequences of all the primers used in this study are listed in Table S2. The sequences were deposited in GenBank; the accession numbers are provided in Table S1.

### 2.2 Reconstruction of the phylogenetic relationship

Three datasets (mtDNA, mtDNA+ncDNA, and single ncDNA) were used to reconstruct the phylogenetic tree. Three congeneric species (*O. amphidoxa, O. thibetari*, and *O. leucopteron*) were used as outgroups, and *O. amphidoxa* and *O. thibetari* are the sister species of *O. szechuana* based on morphometrical analysis. Two methods were used for analysis: Bayesian inference (BI) and maximum likelihood (ML). The partition method we used was based on the different genes. For the BI analysis, a best‐fit model of nucleotide substitution was selected using jModelTest 0.1.1 (Posada, 2008) under the Bayesian information criterion (BIC) (Schwarz, 1978). The BI analysis was carried out in MrBayes 3.1.2 (Ronquist & Huelsenbeck, 2003) under default priors, with each partition unlinked for the parameter estimations. Four Markov chains were run, starting from a random tree and proceeding for 1,000,000 Markov chain Monte Carlo (MCMC) generations, sampling the chains every 100 generations (total 10,000 trees were generated). Two concurrent runs were conducted to verify the results. The first 2,500 trees were discarded as burn‐in samples. The remaining trees were used to compute a majority‐rule consensus tree with posterior probabilities (PP).

The maximum likelihood (ML) analysis was inferred in RAxML v7.2.6 (Stamatakis et al., 2008) with the GTRGAMMAI model for each partition. All the model parameters were estimated during the ML analysis. A rapid bootstrapping algorithm with a random seed value of 12,345 (command ‐f a ‐x 12,345) was applied with 1,000 replicates (Siddall, 2010).

We constructed haplotype networks for combined mtDNA sequence to better visualize the nonbifurcating (multifurcations and reticulations) relationships (Posada & Crandall, 2001). A maximum parsimony method implemented in TCS 1.23 (Clement et al., 2000) was used to draw an unrooted network to evaluate the haplotype relationships for the mtDNA and the ncDNA sequences with 95% parsimoniously plausible branch connections.

### 2.3 Population genetic analyses

To assess how genetic diversity varied across geographic populations, we calculated the following summary statistics based on combined mtDNA sequence datasets. The haplotype diversity (h), the nucleotide diversity (p), and the mean number of pairwise differences were calculated to estimate DNA polymorphism using DnaSP 5.10.01 (Librado & Rozas, 2009). Analysis of molecular variance (AMOVA) and *F*_ST_ calculations were performed using Arlequin 3.5 (Excoffier & Lischer, 2010) with 10,000 permutations, and these analyses were based only on populations that contained more than three individuals.

### 2.4 Estimate of divergence time

The maximum clade credibility tree from divergence‐time‐rooted phylogenetic analyses was estimated using BEAST 1.8.0 (Drummond & Rambaut, 2007) based on the mtDNA dataset. Although the use of a molecular clock as the only way to calibrate the divergence time of phylogenetic trees is controversial, it does provide a method for estimating the approximate divergence time when no other calibration information, such as fossil or geological evidence, is available (López‐López et al., 2015; Maekawa et al., 2001). Thus, the widely accepted mutation rates for the insect mitochondrial COI gene (0.0115–0.0177 per site per million years, Brower, 1994; Papadopoulou et al., 2010) were adopted, and all other genes were scaled to the COI rate in BEAST. Each gene was assigned a separate unlinked relaxed clock model in the analysis. We ran analyses with a coalescent constant tree prior. Both a strict molecular clock model and an uncorrelated lognormal relaxed molecular clock were attempted, and the model comparison of Bayes factors by Akaike information content (AICM) was carried out in BEAST (Baele et al., 2012). MCMC chains were analyzed for 200 million generations and sampled every 2000 generations. Tracer 1.5.0 was used to verify the posterior distribution and the effective sample sizes (ESSs) from the MCMC output. We used TreeAnnotator in the BEAST package to summarize the tree data with “mean height” and discarded the first 25% of the trees due to the “burn‐in” period, which ended well after the stationarity of the chain likelihood values had been established. The tree and divergence times are displayed in FigTree 1.3.1.

### 2.5 Demographic history

The signatures of the population demographic changes were tested for four lineages based on combined mtDNA genes. Tajima's D (Tajima, 1989) and Fu's Fs (Fu, 1997) statistics were used to assess whether the nucleotide polymorphisms deviated from the expectations under the neutral theory in ARLEQUIN. The Bayesian skyline plots (BSP) were implemented in BEAST and used to estimate the changes in population size through time. For each BSP, the substitution model was selected using jModeltest. The samples were drawn every 1,000 steps for 50 million steps under an uncorrelated lognormal relaxed clock model. The mutation rate was set to 0.0115 and 0.0177 per site per million years. The demographic plots were visualized in Tracer 1.5 with a burn‐in of 20%.

### 2.6 Historical biogeography and ecological niche modeling

An ecological niche model was constructed to estimate the potential distributions of *O. szechuana* during the four periods: the Last Interglacial (LIG, 0.13–0.07 Ma), the LGM, the Middle Holocene (MIH, 0.006 Ma), and the Current, with the goal of modeling the impacts of Pleistocene climatic oscillations on species distribution. The ecological niche model was constructed using the maximum entropy machine learning algorithm in MAXENT (Phillips et al., 2006), which has been shown to perform well compared with alternative modeling methods and is robust for low sample sizes. A distribution was generated using 19 climatic variables from the WorldClim database at 2.5‐min resolution for the current climate (Hijmans et al., 2005), with an estimate based on 80 collecting localities from museum records. The LGM climate data were simulated from two models: the Community Climate System Model (CCSM) and the Model for Interdisciplinary Research on Climate (MIROC). The models were each run in ten replicates with default settings. Binomial tests of omission were conducted by randomly selecting 30% of localities as test data. The output of MAXENT consists of grid maps with each cell having an index of suitability between 0 and 1. The model performance was evaluated by averaging the values for the area under the curve (AUC) for the receiver operating characteristic (ROC) curves over ten replicate runs.

### 2.7 Detection and identification of *Wolbachia*

*Wolbachia* is a bacterial endosymbiont of arthropods, and it is extremely widespread among insects. Detection *Wolbachia* is common within phylogenic studies of insects (Cheng, Jiang, et al., 2019; Cheng, Jiang, Xue, et al., 2016). PCR detection of *Wolbachia* infection was performed by using the primers 81F (5′‐TGGTCCAATAAGTGATGAAGAAAC‐3′) and 691R (5′‐AAAAATTAAACG CTACTCCA‐3′) for the wsp gene (Zhou et al., 1998). Infection with *Wolbachia* of either the “A” or “B” supergroup was differentiated by specific PCR targeting of the ftsZ gene: the primers ftsZAdf (5′‐CTCAAGCACTAGAAAAGTCG‐3′) and ftsZAdr (5′‐TTAGCTCCTTCG CTTACGTG‐3′) for the A supergroup, and the primers ftsZBf (5′‐CGATGCTCAA GGGTTAGAG‐3′) and ftsZBr (5′‐CCACTTAACTGTTTGGTTTG‐3′) for the B supergroup (Werren et al., 1995). By using these ftsZ primer sets, the A super group and the B super group of *Wolbachia* were identified by the presence/absence of the PCR products.

## 3 RESULTS

### 3.1 Phylogenetic relationships and estimation of divergence time

**FIGURE 2:**
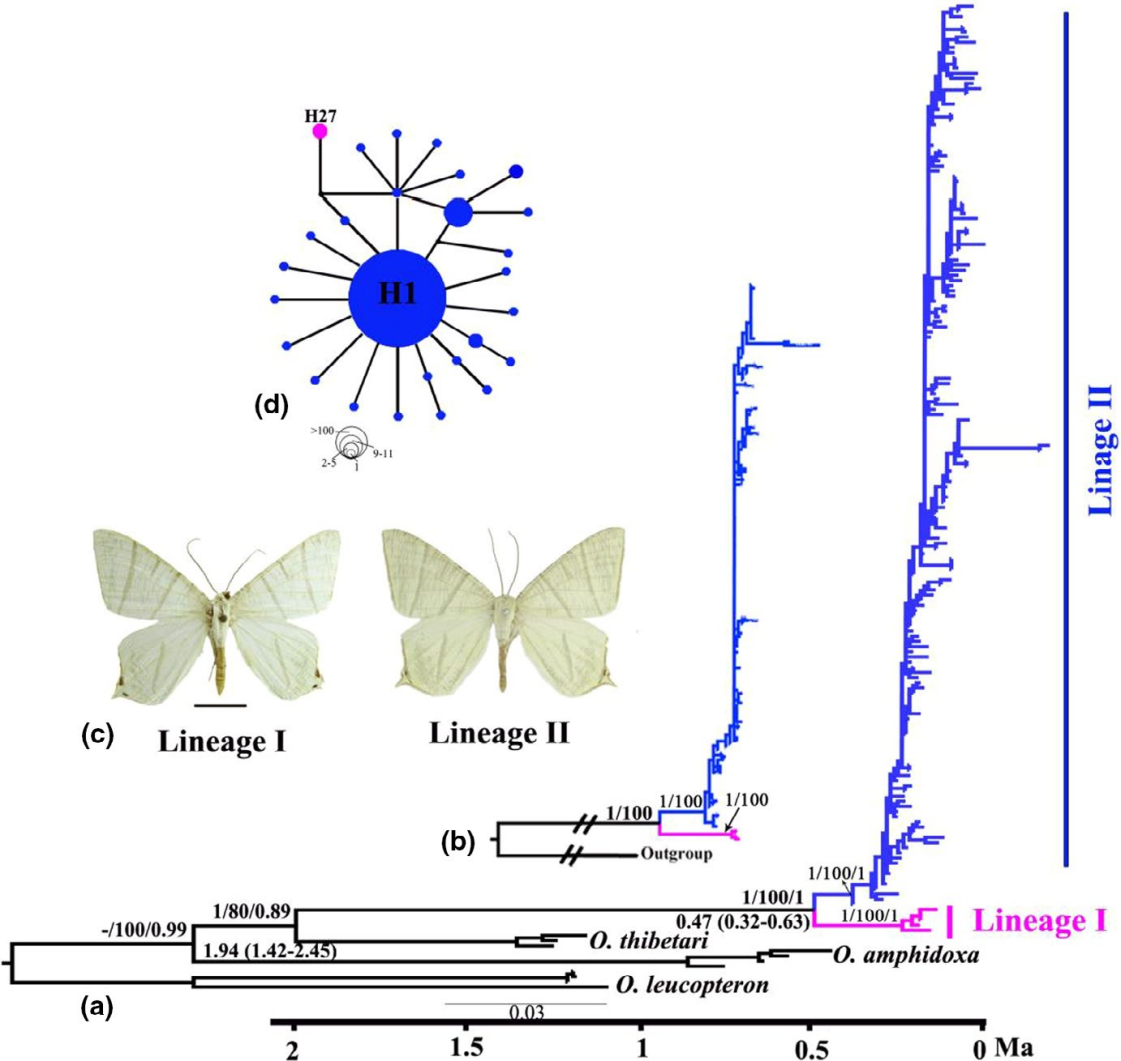
(a) Phylogenetic tree of *O. szechuana* based on combined mtDNA +ncDNA genes. Values on the left of the nodes indicate the posterior probability of the BI/bootstrap and support the ML/posterior probability of BEAST for major clades. Estimates of divergence time with 95% confidence intervals are shown under the nodes. (b) Phylogenetic tree of *O. szechuana* based on combined mtDNA genes. (c) Adults of two lineages. (d) MJ network based on mtDNA for *O. szechuana*. Each circle represents a haplotype, and the size of the circle is proportional to that haplotype's frequency. Dots represent unsampled haplotypes, and dashes represent the corresponding mutational steps. Colors denote lineage membership and are the same as the colors in Figure 1

The phylogenetic analyses of mtDNA genes and combined genes (four mtDNA and three ncDNA genes) (Figure 2) revealed distinct geographical structures in *O. szechuana* and grouped the samples into two reciprocally monophyletic lineages (Lineage I and II) that approximately correspond to the different geographical regions. Lineage I includes individuals from small ranges, including the Shennongjia Mountains in Hubei and the Wushan Mountains in Chongqing. Lineage II includes individuals from throughout the distribution range of *O. szechuana* in China (except the Shennongjia and Wushan mountains) and northern Nepal. The phylogenetic analyses, based on ncDNA genes alone (see Appendix S2), failed to reveal a divergence into two clusters.

The phylogenetic structure as revealed by mtDNA loci was also supported by the networks, which also generated two groups of haplotypes (Figure 2). The polymorphic sites of the mtDNA datasets defined 27 haplotypes, 20 of which were found in a single individual with only one haplotype (H27) belonging to Lineage I. No haplotype was found to be shared by both lineages. The most abundant haplotype (H1) was shared by 121 individuals of Lineage II that were distributed in southern China. The haplotype group corresponding to Lineage II showed a star‐like structure centered on the haplotype, which was the most commonly identified haplotype in the MJ network.

The results showed that a strict molecular clock model performed better than an uncorrelated lognormal relaxed molecular clock model. The Bayes factors used for the model comparison (strict vs. lognormal relaxed) were 31.20 for the combined data set. Therefore, we applied a strict molecular clock model for subsequent analyses. The maximum clade credibility tree of *O. szechuana* in BEAST, based on all mtDNA loci, showed similar results to the BI and ML trees (Figure 2). Divergence time dating indicated that *O. szechuana* diverged from its most closely related species (*O. thibetari*) at approximately 1.94 Ma [1.42–2.45 Ma, 95% highest posterior density (HPD)]. The divergence between Lineage I and Lineage II occurred at approximately 0.47 Ma (0.32–0.63 Ma, 95% HPD).

### 3.2 Genetic diversity

We obtained 3,050 bp mitochondrial genes and 2,533 bp nuclear genes for *O. szechuana*, including COI (627 bp), CYTB (1,005 bp), COII (727 bp), ND5 (691 bp), EF‐1α (964 bp), GAPDH (606 bp), and CAD (963 bp) genes. In the entire mtDNA alignment, 103 sites were variable and 55 were parsimony informative. The values for haplotype (h) and nucleotide diversity (π) of Lineage I were higher (h, 0.833; π, 0.04948) than the values for Lineage II (h, 0.493; π, 0.00223) (Table 1). Based on the combined mtDNA alignment, AMOVA analyses revealed significant genetic differentiation among all populations of *O. szechuana* (*F*_ST_ = 0.154). The average *F_ST_* values for Lineage I and II were 0.25486 and −0.10021, respectively. The most variation was accounted for by variation within populations (84.59%), followed by variation among lineages (26.14%) and variation among populations within lineages (−10.73%).

**TABLE 1:** Summary of genetic diversity, including sample size (*N*), the number of haplotypes (nh), nucleotide diversity (π), haplotype diversity (h), and Fu's Fs and Tajima's D. **p < 0.01

Group	*N*	Nh	Π	h	Fu's Fs	Tajima's D
I	5	1	0.04948	0.833	0.006	1.08976
II	186	26	0.00223	0.493	−31.081**	−2.36978**

### 3.3 Demographic history

**FIGURE 3:**
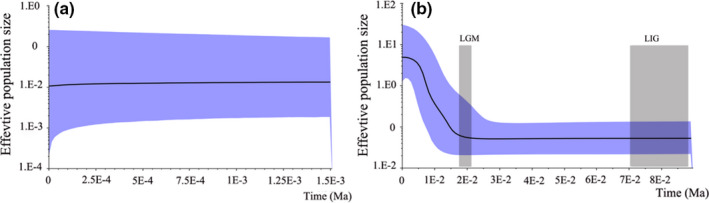
Bayesian skyline plots for two lineages of *O. szechuana*. LGM: the Last Glacial Maximum; LIG: Last Interglacial

Two lineages revealed different demographic models under climatic fluctuations (Figure 3). Lineage I was positive yet not significant, whereas Lineage II was negative but had significant Tajima's D and Fu's Fs values (Table 1). The BSP analyses rejected population stability in Lineage II but could not reject population stability in Lineage I (Figure 3). Recent expansion within Lineage II occurred after the LGM.

### 3.4 Historical distribution change

**FIGURE 4:**
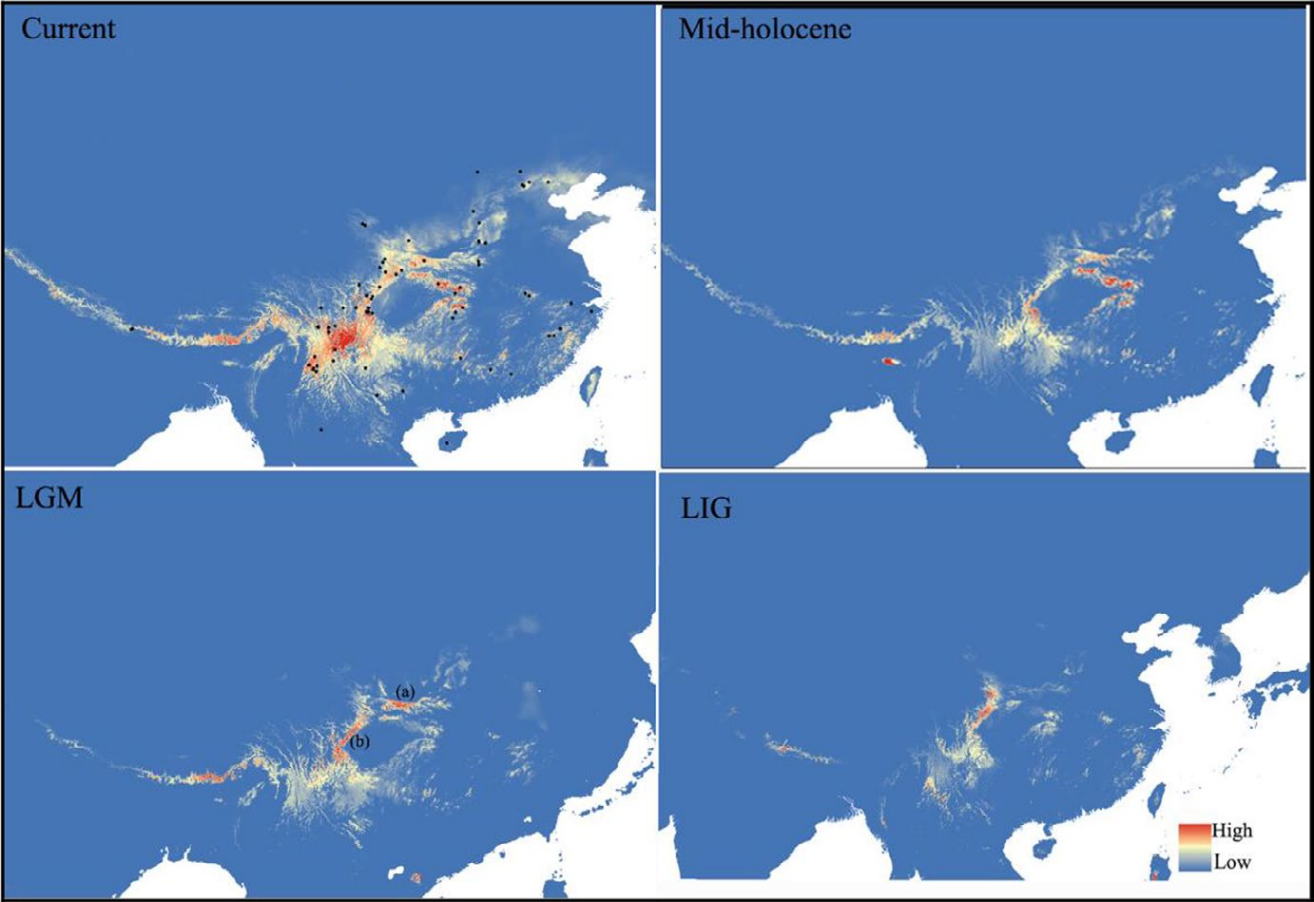
Ecological niche modeling for *O. szechuana* during four periods (LIG, LGM, MIH, and Current). Circles represent the localities used to build the ecological niche model. Different colors correspond to different fitting indices. Low is in blue and high is in red

The ecological niche model for *O. szechuana* (Figure 4) had a high predictive power with an average training AUC of 0.961, 0.969, 0.970, and 0.993. The binomial probabilities (*p* ≪ 0.0001) for the eleven common thresholds also showed that the predictions were substantially better than those obtained using a random model. The respective present‐day distributions of *O. szechuana* were larger than the potential distributions during the other three periods, which means that these two lineages underwent one expansion after the glacial period. The most adaptive distributions of *O. szechuana* during the LGM and Mid‐Holocene periods are around the Sichuan Basin, especially the distribution of Lineage I. According to results for the LGM, two possible refugia (a and b) were revealed: the Qinling Mountains and the western edge of the Sichuan Basin or the easternmost Hengduan Mountains mainly including the Emei, Hailuogou, and Xiling Snow mountains, among others.

### 3.5 Detection and Identification of *Wolbachia*

**FIGURE 5:**
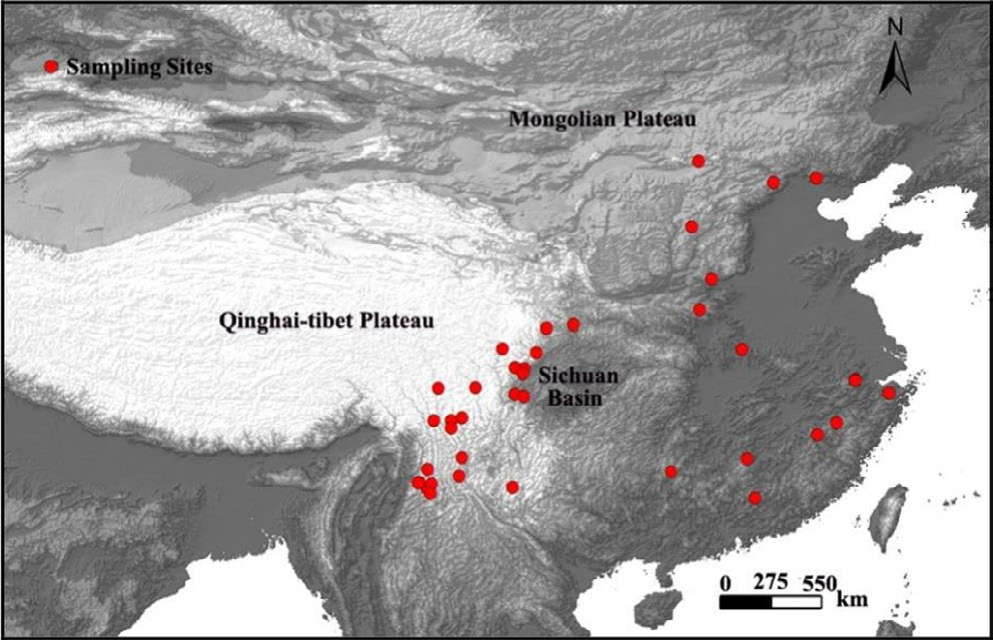
Distribution map of the *Wolbachia* infection for *O. szechuana*

Of the 177 samples (dry samples were excluded) of *O. szechuana* examined by diagnostic PCR, 107 were positive for *Wolbachia,* including all samples in the B supergroup. The frequency was 60.45%. All the infected samples belonged to Lineage II. When data for the *Wolbachia* infection was mapped for the sampling sites (Figure 5), infected populations were distributed throughout most of China.

## 4 DISCUSSION

### 4.1 Evolutionary history of *O. szechuana* and identification of refugia

For *O. szechuana*, the phylogenetic analyses we performed support the reciprocal monophyly of two major lineages, Lineage I and II (Figure 2), although the single ncDNA gene failed to distinguish them (Figure S1). This result may be because the ncDNA genes have fewer variable sites and a slower evolutionary rate than mtDNA genes (Miyata et al., 1982; Sunnucks, 2000), a phenomenon which was also observed in other moth taxa (Cheng, Jiang, Yang, et al., 2016; Cheng et al., 2017; Liu et al., 2016). The two lineages are allopatric (Figure 1). Lineage I is restricted to the Shennongjia and Wushan mountains, whereas Lineage II is distributed throughout the range of the species, across most of China and northern Nepal.

*O. thibetaria,* distributed in the Hengduan‐Himalaya Mountains and adjacent areas (Scoble, 1999; Scoble & Hausmann, 2007), has a smaller distinguished range than the widely distributed *O. szechuana*. In fact, such an inner‐ versus. outer‐Hengduan pattern was already observed (Cheng, Jiang, et al., 2019; Cheng, Jiang, Yang, et al., 2016; Wang et al., 2013). According to the divergence time results (Figure 2), *O. szechuana* diverged from its sister species (*O. thibetaria*) at approximately 1.94 Ma (1.42–2.45 Ma, 95% HPD). During this period, multiple differentiation events of endemic species in the Hengduan Mountains occurred (Fan et al., 2012; Luo et al., 2004; Qu et al., 2006). There is evidence showing that current genetic patterns of intraspecific diversity around the Himalaya‐Hengduan Mountains were more substantially shaped by climatic fluctuations during the Quaternary rather than orogenesis (Mosbrugger et al., 2018; Wan et al., 2016). When glaciations occurred, *O. szechuana* and *O. thibetaria* were restricted at different glacial refugia. Gene flow was interrupted allowing the two species to diverge. After this divergence, *O. szechuana* colonized more areas, even including parts of Hengduan Mountains, whereas *O. thibetaria* remained restricted to Hengduan‐Himalaya Mountains.

In addition, two lineages of *O. szechuana* diverged at ~0.47 Ma (0.32–0.63 Ma, 95% HPD), which directly follows the Naynayxungla Glaciation, or the maximum Pleistocene glaciation (Wu et al., 2002; Zheng et al., 2002). In fact, the Naynayxungla glaciation has been considered an important event in the evolutionary history of plants (Qiu et al., 2009), birds (Qu et al., 2012), and moths (Cheng, Jiang, Yang, et al., 2016). In previous studies, central China (mainly Hubei, Hunan Provinces) was shown to be at least partially covered with ice during Quaternary glaciations (Li et al., 1991; Liu et al., 2002; Shi et al., 2006) and taking their distribution and divergence time into account, allopatric differentiation in different refugia during glacial periods may be the most plausible explanation for the divergence of these two lineages. The Naynayxungla glaciation has been considered an important event in the evolutionary history of plants (Qiu et al., 2009), birds (Qu et al., 2012), and moths (Cheng, Jiang, Yang, et al., 2016).

An accurate identification of glacial refugia is a high priority for conservation because these are key areas for investigating the persistence and evolution of biodiversity (López‐Pujol et al., 2011; Shen et al., 2002; Tzedakis et al., 2002). For Lineage I of *O. szechuana*, population size was stable, and recent expansion did not occur (Figure 3). Considering the restricted distribution and relatively high genetic diversity (Figure 1 and Table 1), we infer that the refugia were located in the Shennongjia–Wushan Mountains. However, the star‐shaped network, the negative yet significant Fu’Fs and Tajima's analyses, and the BSP and ecological niche modeling results all support that Lineage II has gone recent expansion after the LGM. According to the results of ecological niche modeling results, there are two possible refugia: (a) the Qinling Mountains and (b) the western edge of the Sichuan Basin or the easternmost part of the Hengduan Mountains, including the Emei, Hailuogou, and Xiling Snow Mountains. Thus, our results align with those of many earlier studies, the Qinling Mountains have been confirmed as a refugium area in multiple groups, including frogs (Wang et al., 2012), plants (Zhao et al., 2013; Zhou et al., 2010), mammals (Li et al., 2018), and birds (Wu et al., 2017), similarly to the Hengduan Mountains (Li et al., 2017; Wang et al., 2013; Wu et al., 2017).

### 4.2 Cryptic refugia of the Shennongjia–Wushan Mountains

The Shennongjia–Wushan Mountains are located in the northwest of the Sichuan Basin and are adjacent to the Daba Mountains at the contact zone between the plains and foothill regions of eastern China and the mountainous region of central China, and are also situated along a zone of climate transition (Ying, 2001; Yu et al., 2019). The Shennongjia Mountains are well known for hosting multiple relict species (such as Chinese dove tree and snub‐nosed monkey) and have also been considered as one of the three centers hosting the greatest biodiversity of seed plants in China (Ying, 2001; Zhang et al., 2018). However, compared with the Hengduan and Qinling mountains, the Shennongjia–Wushan Mountains are less known as a glacial refugium and are often underestimated due to research focusing on groups with weak dispersal ability, such as plants (Lei et al., 2012; Yao et al., 2012; Zhang et al., 2015), frogs (Qiao et al., 2018), and crabs (Fang et al., 2013). There are in fact only few studies tackling taxa with a potentially stronger dispersal ability, such as insects, birds, and mammals. Our phylogeographical study thus bridges this gap by confirming the occurrence of a refugium in the Shennongjia–Wushan Mountains for *O. szechuana*, a moth with a wide distribution range and a certain dispersal ability. Across the whole distribution range of *O. szechuana*, there are larger and more topographically complex mountain regions, but only the Shennongjia–Wushan Mountains hosted a private lineage, which confirmed the uniqueness of this region. The extent of glaciation during the Quaternary glaciation is not fully understood yet (Muellner‐Riehl, 2019), and there is no precise overview of the extent of glaciation in the Shennongjia–Wushan Mountains available. However, it is believed that glaciers in the Hengduan Mountains were restricted to high elevations, above 2,000 m. In any case, regardless of the extent of ice cover, the biota of central China are likely to have experienced vertical displacement during glaciations, probably at the source of the patterns we observe today (Li et al., 1991; Liu et al., 2002; Lei et al., 2015; Muellner‐Riehl, 2019; Ren et al., 2020; Qu et al., 2014). Connecting with Yangtze River and its tributaries, the valleys of the Shennongjia–Wushan Mountains may have remained relatively ice‐free and characterized by relatively warmer and more stable environmental conditions, thus served as refugia as argued for plants (Lei et al., 2012; Yao et al., 2012; Zhang et al., 2015). As key areas for investigating the persistence and evolution of biodiversity, the glacial refugia of the Shennongjia–Wushan Mountains should be highly prioritized for conservation (López‐Pujol et al., 2011; Shen et al., 2002; Tzedakis et al., 2002).

### 4.3 Comparison of the structure of two lineages of *O. szechuana*

Compared with Lineage I which is restricted to the Shennongjia and Wushan mountains, populations and individuals of Lineage II harbor low genetic differentiation and the lineage is widely distributed across most of China. We did not find any other studies where such a difference was found between the Shennongjia and Wushan mountains and all other surrounding areas. This is in fact surprising because the Shennongjia and Wushan mountains are way less complex topographically than the Hengduan Mountains, where more evidence for refugia and species or genetic endemicity would be expected. There are several potential explanations for this phenomenon. First, the Shennongjia and Wushan mountains may have been more isolated throughout their history than previously thought, preventing populations from expending to other neighboring mountain ranges. In contrast, the refugia of Lineage II might have been better interconnected by corridors providing better dispersal routes to distant areas. Second, the expansion of Lineage II may have occurred very rapidly in recent times but a significant while after its divergence from Lineage I (0.4 Ma), and indeed the BSP results support the claim that the recent expansion occurred after the LGM (0.021 Ma, Figure 3). This rapid expansion, probably aided by the strong dispersal ability of this flying species, would have filled the available ecological niches throughout, preventing the successful expansion of Lineage I. Finally, our interpretations are supported by the fact that the rate of *Wolbachia* infections is high in Lineage II throughout its range and null in Lineage I (Figure 5). Although harmful, previous studies have shown that *Wolbachia* infection can improve the adaptation potential of its hosts (Gong et al., 2002; Kambris et al., 2009; Walker et al., 2011) and in some cases, even increase its dispersal ability (e.g., *Trichogramma cordubensis*; Silva et al., 2000). Although this does not constitute a direct evidence, the high frequency of *Wolbachia* infections in Lineage II may have allowed these lineages to expand faster in comparison with Lineage I.

## 5 CONCLUSION

By associating molecular data with the geological and climatic backgrounds, our study is the first to confirm that the area of the Shennongjia–Wushan Mountains acted as glacial refugium during the climate oscillations of the Quaternary. Our results are mirrored by further analysis on *Wolbachia*, a well‐known bacterial endosymbiont of arthropods.

## CONFLICT OF INTEREST

The authors declare that they have no conflict of interest.

## AUTHOR CONTRIBUTION

**Rui Cheng:** Data curation (equal); Formal analysis (equal); Funding acquisition (equal); Methodology (equal); Resources (equal); Software (equal); Writing‐original draft (equal); Writing‐review & editing (equal). **Hongxiang Han:** Funding acquisition (equal); Investigation (equal); Project administration (equal); Writing‐review & editing (equal). **DaYong Xue:** Data curation (equal); Formal analysis (equal); Funding acquisition (equal); Project administration (equal); Writing‐review & editing (equal). **Chaodong Zhu:** Funding acquisition (equal); Investigation (equal); Project administration (equal). **Nan Jiang:** Data curation (equal); Formal analysis (equal); Funding acquisition (equal); Methodology (equal); Project administration (equal); Resources (equal); Software (equal); Writing‐original draft (equal); Writing‐review & editing (equal).

## Supporting information

Supplementary Material

## Data Availability

Total DNA sequences were deposited in GenBank; the accession numbers are provided in Table S1.
