# Supplementary material for: Shennongjia–Wushan Mountains—One cryptic glacial refugium introduced by the phylogeographical study of the Geometridae moth Ourapteryx szechuana Wehrli
Source: Ecol Evol. 2021 Jun 21;11(15):10066–76. doi: 10.1002/ece3.7794 (PMC8328460; doi:10.1002/ece3.7794)

*Ecology and Evolution*

**SUPPORTING INFORMATION**

**Shennongjia-Wushan Mountains—one cryptic glacial refugium introduced by the phylogeographical study of the Geometridae moth *Ourapteryx szechuana* Wehrli**

Rui Cheng, Hongxiang Han, Dayong Xue, Nan Jiang

Table S1 Sampling information and the GenBank accession numbers of *Ourapteryx szechuana* used in the study.

| Voucher | Code | Location | Collectors | Country | Altitudes | Coi | Nd5 | Cytb | COII | Ef-1a | GAPDH | CAD |
| --- | --- | --- | --- | --- | --- | --- | --- | --- | --- | --- | --- | --- |
| IOZ LEP M 13581 | TC | Houqiao Town, Yunnan | Xinxin Li | China | 1553 | MG907918 | MG909373 | MG908395 |  | MG908797 | MG909033 | MK421025 |
| IOZ LEP M 13582 | TC | Houqiao Town, Yunnan | Xinxin Li | China | 1553 | MG907919 | MG909374 | MG908396 |  | MG908798 | MK420771 | MK421026 |
| IOZ LEP M 13587 | TC | Tengchong wetland, Yunnan | Xinxin Li | China | 1697 | MG907920 | MG909375 | MG908397 |  | MG908799 | MG909034 | MK421027 |
| IOZ LEP M 13589 | TC | Tengchong wetland, Yunnan | Xinxin Li | China | 1697 | MG907921 | MG909376 | MG908398 |  | MG908800 | MK420772 | MK421028 |
| IOZ LEP M 13590 | TC | Tengchong wetland, Yunnan | Xinxin Li | China | 1697 | MG907922 | MG909377 | MG908399 | MK420903 | MG908801 | MK420773 | MK421029 |
| IOZ LEP M 13595 | MEK | Ma’erkang City, Sichuan | Xiaodan Pan | China | 2662 | MG907923 | MG909378 | MG908400 |  | MG908802 | MK420774 | MK421030 |
| IOZ LEP M 13596 | MEK | Ma’erkang City, Sichuan | Xiaodan Pan | China | 2662 | MG907924 | MG909379 | MG908401 |  | MG908803 | MK420775 | MK421031 |
| IOZ LEP M 13598 | MEK | Ma’erkang City, Sichuan | Xiaodan Pan | China | 2662 | MG907925 | MG909380 | MG908402 | MK420904 |  | MK420776 | MK421032 |
| IOZ LEP M 13599 | MEK | Ma’erkang City, Sichuan | Xiaodan Pan | China | 2662 | MG907926 | MG909381 | MG908403 | MK420905 | MG908804 | MK420777 |  |
| IOZ LEP M 14066 | CM | Cang Mountain, Yunnan | Xiaodan Pan | China | 2226 | MG907927 | MG909382 | MG908404 | MK420906 |  | MK420778 | MK421033 |
| IOZ LEP M 14100 | TC | Heinitang, Yunnan | Xiaodan Pan | China | 1824 | MG907928 |  | MG908405 | MK420907 | MG908805 | MK420779 | MK421034 |
| IOZ LEP M 14250 | PM | Pianma Town, Yunnan | Xinxin Li | China | 1980 | MG907929 | MG909383 | MG908406 | MK420908 | MG908806 | MK420780 | MK421035 |
| IOZ LEP M 14518 | GZ | Gezan Town, Yunnan | Xinxin Li | China | 3141 | MG907930 |  | MG908407 | MK420909 | MG908807 | MK420781 | MK421036 |
| IOZ LEP M 14519 | GZ | Gezan Town, Yunnan | Xinxin Li | China | 3141 | MG907931 | MG909384 | MG908408 | MK420910 | MG908808 | MK420782 | MK421037 |
| IOZ LEP M 14564 | SV | E’chu Village, Daocheng, Sichuan | Xinxin Li | China | 2880 | MG907932 | MG909385 | MG908409 | MK420911 | MG908809 | MK420783 | MK421038 |
| IOZ LEP M 14565 | SV | E’chu Village, Daocheng, Sichuan | Xinxin Li | China | 2880 | MG907933 | MG909386 | MG908410 | MK420912 | MG908810 | MK420784 | MK421039 |
| IOZ LEP M 14596 | ZD | Zhongdui Village, Daocheng, Sichuan | Xinxin Li | China | 2880 |  |  | MG908411 | MK420913 | MG908811 | MK420785 | MK421040 |
| IOZ LEP M 14638 | BT | Batang, Sichuan | Xinxin Li | China | 2662 | MG907934 | MG909387 | MG908412 | MK420914 | MG908812 | MG909035 | MK421041 |
| IOZ LEP M 14669 | YJ | Yajiang, Sichuan | Xinxin Li | China | 3340 | MG907935 | MG909388 | MG908413 | MK420915 | MG908813 | MK420786 | MK421042 |
| IOZ LEP M 14673 | YJ | Yajiang, Sichuan | Xinxin Li Xinxin Li | China | 3340 | MG907936 | MG909389 | MG908414 | MK420916 | MG908814 | MK420787 | MK421043 |
| IOZ LEP M 16650 | JZG | Jiuzhaigou, Sichuan | Xinxin Li | China | 2045 | MG907937 | MG909390 | MG908415 | MK420917 | MG908815 |  |  |
| IOZ LEP M 16660 | LZB | Liziba, Gansu | Xiaodan Pan | China | 1971 | MG907938 | MG909391 | MG908416 | MK420918 | MG908816 | MK420788 | MK421044 |
| IOZ LEP M 16661 | LZB | Liziba, Gansu | Xiaodan Pan | China | 1971 | MG907939 | MG909392 | MG908417 | MK420919 | MG908817 | MK420789 | MK421045 |
| IOZ LEP M 16672 | LZB | Liziba, Gansu | Xiaodan Pan | China | 1971 | MG907940 | MG909393 | MG908418 | MK420920 | MG908818 | MK420790 | MK421046 |
| IOZ LEP M 16674 | LZB | Liziba, Gansu | Xiaodan Pan | China | 1971 | MG907941 | MG909394 | MG908419 | MK420921 | MG908819 | MK420791 | MK421047 |
| IOZ LEP M 16676 | LZB | Liziba, Gansu | Xiaodan Pan | China | 1971 | MG907942 | MG909395 | MG908420 | MK420922 | MG908820 | MK420792 | MK421048 |
| IOZ LEP M 16686 | YJ | Yajiang, Sichuan | Xiaodan Pan | China | 3340 | MG907943 | MG909396 | MG908421 | MK420923 | MG908821 | MK420793 | MK421049 |
| IOZ LEP M 16689 | YJ | Yajiang, Sichuan | Xiaodan Pan | China | 3340 | MG907944 | MG909397 | MG908422 | MK420924 | MG908822 | MK420794 | MK421050 |
| IOZ LEP M 16697 | YJ | Yajiang, Sichuan | Xiaodan Pan | China | 3340 | MG907945 | MG909398 | MG908423 | MK420925 | MG908823 | MK420795 | MK421051 |
| IOZ LEP M 16736 | XL | Siguniang Mountain, Sichuan | Xinxin Li | China | 3264 | MG907946 | MG909399 | MG908424 | MK420926 | MG908824 | MK420796 | MK421052 |
| IOZ LEP M 16738 | XL | Siguniang Mountain, Sichuan | Xinxin Li | China | 3264 | MG907947 | MG909400 | MG908425 | MK420927 | MG908825 | MK420797 | MK421053 |
| IOZ LEP M 16743 | XL | Siguniang Mountain, Sichuan | Xinxin Li | China | 3264 | MG907948 | MG909401 | MG908426 | MK420928 |  | MK420798 | MK421054 |
| IOZ LEP M 16746 | XL | Siguniang Mountain, Sichuan | Xinxin Li | China | 3264 | MG907949 | MG909402 | MG908427 | MK420929 | MG908826 | MK420799 | MK421055 |
| IOZ LEP M 16751 | XL | Siguniang Mountain, Sichuan | Xinxin Li | China | 3264 | MG907950 | MG909403 | MG908428 | MK420930 |  | MK420800 |  |
| IOZ LEP M 16774 | LZB | Liziba, Gansu | Xinxin Li | China | 1971 | MG907951 | MG909404 | MG908429 |  | MG908656 |  |  |
| IOZ LEP M 16782 | BT | Batang, Sichuan | Xinxin Li | China | 2662 | MG907952 | MG909405 | MG908430 | MK420931 | MG908827 | MK420801 | MK421056 |
| IOZ LEP M 16893 | PM | Pianma Town, Yunnan | Xinxin Li | China | 1980 | MG907953 | MG909406 | MG908431 | MK420932 |  | MK420802 |  |
| IOZ LEP M 16898 | PM | Pianma Town, Yunnan | Xinxin Li | China | 1980 | MG907954 | MG909407 | MG908432 |  | MG908828 | MG909036 | MK421057 |
| IOZ LEP M 16899 | PM | Pianma Town, Yunnan | Xinxin Li | China | 1980 | MG907955 | MG909408 | MG908433 |  |  |  |  |
| IOZ LEP M 16926 | DQ | Gongziding Village, Yunnan | Xinxin Li | China | 2914 | MG907956 | MG909409 | MG908434 | MK420933 | MG908829 | MK420803 | MK421058 |
| IOZ LEP M 16927 | DQ | Gongziding Village, Yunnan | Xinxin Li | China | 2914 | MG907957 | MG909410 | MG908435 | MK420934 | MG908830 | MK420804 | MK421059 |
| IOZ LEP M 16928 | DQ | Gongziding Village, Yunnan | Xinxin Li | China | 2914 | MG907958 | MG909411 | MG908436 |  | MG908831 | MK420805 | MK421060 |
| IOZ LEP M 16930 | DQ | Gongziding Village, Yunnan | Xinxin Li | China | 2914 | MG907959 | MG909412 | MG908437 | MK420935 | MG908832 | MK420806 | MK421061 |
| IOZ LEP M 16931 | DQ | Gongziding Village, Yunnan | Xinxin Li | China | 2914 | MG907960 | MG909413 | MG908438 | MK420936 | MG908833 | MK420807 | MK421062 |
| IOZ LEP M 16934 | GZ | Gezan Town, Yunnan | Xinxin Li | China | 3141 | MG907961 | MG909414 | MG908439 | MK420937 | MG908834 | MK420808 | MK421063 |
| IOZ LEP M 16935 | GZ | Gezan Town, Yunnan | Xinxin Li | China | 3141 | MG907962 | MG909415 | MG908440 | MK420938 | MG908835 | MK420809 | MK421064 |
| IOZ LEP M 16943 | GZ | Gezan Town, Yunnan | Xinxin Li | China | 3141 | MG907963 | MG909416 | MG908441 | MK420939 | MG908836 | MK420810 | MK421065 |
| IOZ LEP M 16944 | GZ | Gezan Town, Yunnan | Xinxin Li | China | 3141 | MG907964 | MG909417 | MG908442 | MK420940 | MG908837 | MK420811 | MK421066 |
| IOZ LEP M 16949 | GZ | Gezan Town, Yunnan | Xiaodan Pan | China | 3141 |  | MG909418 | MG908443 | MK420941 |  | MK420812 | MK421067 |
| IOZ LEP M 16952 | GZ | Gezan Town, Yunnan | Xiaodan Pan | China | 3141 | MG907965 | MG909419 | MG908444 |  | MG908838 | MK420813 | MK421068 |
| IOZ LEP M 16956 | SV | E’chu Village, Daocheng, Sichuan | Xiaodan Pan | China | 2880 | MG907966 | MG909420 | MG908445 |  | MG908839 | MK420814 | MK421069 |
| IOZ LEP M 16957 | SV | E’chu Village, Daocheng, Sichuan | Xiaodan Pan | China | 2880 | MG907967 | MG909421 | MG908446 | MK420942 | MG908840 | MK420815 | MK421070 |
| IOZ LEP M 16958 | SV | E’chu Village, Daocheng, Sichuan | Xiaodan Pan | China | 2880 | MG907968 |  | MG908447 | MK420943 | MG908841 |  | MK421071 |
| IOZ LEP M 16960 | ZD | Zhongdui Village, Daocheng, Sichuan | Xiaodan Pan | China | 2880 | MG907969 | MG909422 | MG908448 | MK420944 | MG908842 | MK420816 |  |
| IOZ LEP M 16961 | ZD | Zhongdui Village, Daocheng, Sichuan | Xiaodan Pan | China | 2880 | MG907970 | MG909423 | MG908449 | MK420945 |  | MK420817 |  |
| IOZ LEP M 16986 | YB | Yangba, Gansu | Dayong Xue | China | 1020 | MG907971 | MG909424 | MG908450 |  | MG908843 | MK420818 |  |
| IOZ LEP M 16991 | YB | Yangba, Gansu | Dayong Xue | China | 1020 | MG907972 | MG909425 | MG908451 |  | MG908844 | MK420819 |  |
| IOZ LEP M 16994 | YB | Yangba, Gansu | Xiaoshuang Ban | China | 1020 | MG907973 | MG909426 | MG908452 |  | MG908845 | MK420820 |  |
| IOZ LEP M 17736 | Nepal | Matathati Village | Rui Chen | Nepal | 1180 | MG907974 | MG909427 | MG908453 |  |  |  |  |
| IOZ LEP M 17742 | Nepal | Matathati Village | Rui Chen | Nepal | 1180 | MG907975 | MG909428 | MG908454 |  |  |  |  |
| IOZ LEP M 17743 | Nepal | Matathati Village | Rui Chen | Nepal | 1180 | MG907976 | MG909429 | MG908455 | MK420946 |  |  |  |
| IOZ LEP M 19955 | MX | Mao County, Sichuan | Xinxin Li | China | 1848 | MG907977 | MG909430 | MG908456 |  | MG908846 |  | MK421074 |
| IOZ LEP M 19956 | MX | Mao County, Sichuan | Xinxin Li | China | 1848 | MG907978 | MG909431 | MG908457 |  | MG908847 | MK420830 | MK421075 |
| IOZ LEP M 19957 | MX | Mao County, Sichuan | Xinxin Li | China | 1848 | MG907979 | MG909432 | MG908458 | MK420954 |  | MK420831 | MK421076 |
| IOZ LEP M 19959 | MX | Mao County, Sichuan | Xinxin Li | China | 1848 | MG907980 | MG909433 | MG908459 |  | MG908848 |  | MK421077 |
| IOZ LEP M 21548 | DQ | Dawei Mountain, Yunnan | Lanbin Xiang | China | 2043 | MG907981 | MG909434 | MG908460 | MK420956 | MG908849 | MK420833 | MK421079 |
| IOZ LEP M 22193 | DQ | Deqin City, Yunnan | Lanbin Xiang | China | 3306 | MG907982 | MG909435 | MG908461 |  | MG908850 | MG909037 | MK421081 |
| IOZ LEP M 22194 | DQ | Deqin City, Yunnan | Xiaoshuang Ban | China | 3306 | MG907983 | MG909436 | MG908462 |  | MG908851 | MG909038 | MK421082 |
| IOZ LEP M 23113 | EMM | Wawu Mountain, Sichuan | Le Cui | China | 1147 | MG907984 | MG909437 | MG908463 | MK420958 |  | MG909039 |  |
| IOZ LEP M 23904 | GEG | Goose Gorge, Tanchang City, Gansu | Rui Cheng | China | 2045 | MG907985 | MG909438 | MG908464 | MK42095 | MG908852 | MG909040 | MK421083 |
| IOZ LEP M 26067 | YJ | Kangding City, Sichuan | Henan Li | China | 2581 | MG907986 | MG909439 | MG908465 |  | MG908853 | MG909041 | MK421085 |
| IOZ LEP M 26070 | YJ | Kangding City, Sichuan | Henan Li | China | 2581 | MG907987 | MG909440 | MG908466 | MK420962 |  |  |  |
| IOZ LEP M 26072 | YJ | Kangding City, Sichuan | Le Cui | China | 2581 | MG907988 | MG909441 | MG908467 | MK420963 | MG908854 | MG909042 | MK421086 |
| IOZ LEP M 26073 | YJ | Kangding City, Sichuan | Le Cui | China | 2581 | MG907989 | MG909442 | MG908468 |  | MG908855 | MG909043 | MK421087 |
| IOZ LEP M 26074 | YJ | Kangding City, Sichuan | Le Cui | China | 2581 | MG907990 | MG909443 | MG908469 |  | MG908856 | MG909044 |  |
| IOZ LEP M 26076 | DQ | Dawei Mountain, Yunnan | Xiaoshuang Ban | China | 2043 | MG907991 |  | MG908470 | MK420964 |  |  |  |
| IOZ LEP M 7563 | CM | Cang Mountain, Yunnan | Shuxian Liu | China | 2226 | MG907992 |  | MG908471 | MK420872 | MG908857 | MK420737 | MK420989 |
| IOZ LEP M 7564 | CM | Cang Mountain, Yunnan | Shuxian Liu | China | 2226 | MG907993 | MG909444 | MG908472 |  | MG908858 |  |  |
| IOZ LEP M 7603 | XL | Sanjiang, Sichuan | Rui Cheng | China | 1349 | MG907994 | MG909445 | MG908473 |  | MG908859 | MK420738 | MK420990 |
| IOZ LEP M 7605 | XL | Sanjiang, Sichuan | Rui Cheng | China | 1349 | MG907995 | MG909446 | MG908474 | MK420873 | MG908860 | MK420739 | MK420991 |
| IOZ LEP M 7607 | XL | Sanjiang, Sichuan | Rui Cheng | China | 1349 | MG907996 | MG909447 | MG908475 |  | MG908861 |  | MK420992 |
| IOZ LEP M 7608 | XL | Sanjiang, Sichuan | Rui Cheng | China | 1349 | MG907997 | MG909448 | MG908476 |  | MG908862 |  | MK420993 |
| IOZ LEP M 7642 | EMS | Linggongli, E’Mei Mountain, Sichuan | Rui Cheng | China | 1288 | MG907998 | MG909449 | MG908477 | MK420878 | MG908863 | MK420744 | MK420997 |
| IOZ LEP M 7643 | EMS | Linggongli, E’Mei Mountain, Sichuan | Rui Cheng | China | 1288 | MG907999 | MG909450 | MG908478 | MK420879 MK42087 | MG908864 | MK420745 | MK420998 |
| IOZ LEP M 7646 | EMS | Linggongli, E’Mei Mountain, Sichuan | Rui Cheng | China | 1288 | MG908000 | MG909451 | MG908479 | MK420880 | MG908865 | MK420746 | MK420999 |
| IOZ LEP M 7649 | EMS | Linggongli, E’Mei Mountain, Sichuan | Rui Cheng | China | 1288 | MG908001 | MG909452 | MG908480 | MK420881 | MG908866 |  | MK421000 |
| IOZ LEP M 7650 | SV | Xiangcheng, Sichuan | Rui Cheng | China | 2841 | MG908002 | MG909453 | MG908481 | MK420882 MK42088 | MG908867 | MK420747 |  |
| IOZ LEP M 7651 | SV | Shangri-la valley, Yunnan | Rui Cheng | China | 3029 | MG908003 | MG909454 | MG908482 | MK420883 | MG908868 |  | MK421001 |
| IOZ LEP M 7652 | SV | Shangri-la valley, Yunnan | Rui Cheng | China | 3029 | MG908004 | MG909455 | MG908483 |  | MG908869 | MK420748 | MK421002 |
| IOZ LEP M 7653 | SV | Shangri-la valley, Yunnan | Rui Cheng | China | 3029 | MG908005 | MG909456 | MG908484 |  | MG908870 |  | MK421003 |
| IOZ LEP M 7654 | XL | Xilingxueshan, Sichuan | Rui Cheng | China | 1352 | MG908006 | MG909457 | MG908485 | MK420884 | MG908871 |  | MK421004 |
| IOZ LEP M 7661 | SV | Shangri-la valley, Yunnan | Rui Cheng | China | 3029 | MG908007 | MG909458 | MG908486 | MK420885 | MG908872 | MK420749 | MK421005 |
| IOZ LEP M 7665 | ZD | Zhengding Village, Yunnan | Xinxin Li | China | 1833 | MG908008 | MG909459 | MG908487 | MK420886 | MG908873 |  | MK421006 |
| IOZ LEP M 7666 | ZD | Zhengding Village, Yunnan | Shuxian Liu | China | 1833 | MG908009 | MG909460 | MG908488 | MK420887 | MG908874 |  | MK421007 |
| IOZ LEP M 7777 | TC | Laifeng Mountain, Yunnan | Xiushuai Yang | China | 1700 | MG908010 |  |  |  |  | MK420752 |  |
| IOZ LEP M 7783 | YNAU | Yunnan Agriculture University, Yunnan | Ke Wang | China |  | MG908011 | MG909461 | MG908489 | MK420890 | MG908875 | MK420753 | MK421010 |
| IOZ LEP M 7784 | YNAU | Yunnan Agriculture University, Yunnan | Xiushuai Yang | China |  | MG908012 | MG909462 | MG908490 |  | MG908876 | MK420754 |  |
| IOZ LEP M 9113 | BHL | Baihualing, Yunnan | Xinxin Li | China | 1520 | MG908013 | MG909463 | MG908491 | MK420891 | MG908877 |  | MK421012 |
| IOZ LEP M 9380 | EMS | Linggongli, E’Mei Mountain, Sichuan | Rui Cheng | China | 1288 |  | MG909464 |  | MK420892 | MK420656 | MK420758 | MK421013 |
| IOZ LEP M 9384 | EMS | Linggongli, E’Mei Mountain, Sichuan | Rui Cheng | China | 1288 | MG908014 | MG909465 | MG908492 | MK420893 | MG908878 | MK420759 | MK421014 |
| IOZ LEP M 9465 | EMS | Wuxiangang, E’Mei Mountain, Sichuan | Rui Cheng | China | 650 | MG908015 | MG909466 | MG908493 | MK420894 | MG908879 |  | MK421015 |
| IOZ LEP M 9476 | EMM | Hailuogou, Sichuan | Rui Cheng | China | 1949 | MG908016 | MG909467 | MG908494 |  | MG908880 | MK420760 | MK421016 |
| IOZ LEP M 9530 | SV | Xiangcheng, Sichuan | Rui Cheng | China | 2841 | MG908017 | MG909468 | MG908495 | MK420895 | MG908881 | MK420761 | MK421017 |
| IOZ LEP M 9562 | SV | Xiangcheng, Sichuan | Rui Cheng | China | 2841 | MG908018 | MG909469 | MG908496 | MK420896 | MG908882 | MK420762 | MK421018 |
| IOZ LEP M 9579 | SV | Shangri-la valley, Yunnan | Rui Cheng | China | 3029 | MG908019 | MG909470 | MG908497 |  | MG908883 | MK420763 | MK421019 |
| IOZ LEP M 9580 | SV | Shangri-la valley, Yunnan | Rui Cheng | China | 3029 | MG908020 | MG909471 | MG908498 |  | MG908884 |  |  |
| IOZ LEP M 01236 | TMM | Tianmu Mountain, Zhejiang | Rui Cheng | China | 789 | MK420494 | MK420685 | MK420565 | MK420862 | MK420643 | MK420727 | MK420980 |
| IOZ LEP M 01287 | TMM | Zhonglieci, Tianmu Mountain, Zhejiang | Rui Cheng | China | 363 | MK420495 | MK420686 | MK420566 | MK420863 | MK420644 | MK420728 | MK420981 |
| IOZ LEP M 01394 | TMM | Xianrending, Tianmu Mountain,Zhejiang | Rui Cheng | China | 1506 | MK420496 | MK420687 | MK420567 | MK420864 | MK420645 | MK420729 | MK420982 |
| IOZ LEP M 01442 | TMM | Qianmutian, Tianmu Mountain,Zhejiang | Keji Yan | China | 1330 | MK420497 | MK420688 | MK420568 | MK420865 | MK420646 | MK420730 | MK420983 |
| IOZ LEP M 04008 | TJ | Baxian Mountain,Tianjin | Fuqiang Chen | China |  |  | MK420689 | MK420569 | MK420866 | MK420647 | MK420731 | MK420984 |
| IOZ LEP M 06849 | MEM | Antangping, Mao'er Mountain,Guangxi | Chao Yang | China | 1579 | MK420498 | MK420690 | MK420570 | MK420867 | MK420648 | MK420732 | MK420985 |
| IOZ LEP M 06936 | MEM | Antangping, Mao'er Mountain,Guangxi | Chao Yang | China | 1579 | MK420499 | MK420691 | MK420571 |  |  | MK420733 | MK420986 |
| IOZ LEP M 07552 | JLM | Daqiutian, Jiulian Mountain, Jiangxi | Chaodong Zhu | China |  | MK420500 |  | MK420572 |  |  | MK420734 |  |
| IOZ LEP M 07553 | JLM | Daqiutian, Jiulian Mountain, Jiangxi | Chaodong Zhu | China | 800-1430 | MK420501 | MK420692 | MK420573 |  | MK420649 | MK420735 | MK420987 |
| IOZ LEP M 07554 | JLM | Daqiutian, Jiulian Mountain, Jiangxi | Chaodong Zhu | China | 800-1430 | MK420502 | MK420693 | MK420574 |  |  |  |  |
| IOZ LEP M 07555 | JLM | Daqiutian, Jiulian Mountain, Jiangxi | Chaodong Zhu | China | 800-1430 | MK420503 | MK420694 | MK420575 | MK420868 |  |  |  |
| IOZ LEP M 07556 | JLM | Daqiutian, Jiulian Mountain, Jiangxi | Chaodong Zhu | China | 800-1430 | MK420504 | MK420695 | MK420576 | MK420869 |  |  |  |
| IOZ LEP M 07557 | JLM | Daqiutian, Jiulian Mountain, Jiangxi | Chaodong Zhu | China | 800-1430 | MK420505 | MK420696 | MK420577 | MK420870 | MK420650 | MK420736 | MK420988 |
| IOZ LEP M 07558 | JLM | Daqiutian, Jiulian Mountain, Jiangxi | Chaodong Zhu | China | 800-1430 | MK420506 | MK420697 | MK420578 | MK420861 |  |  |  |
| IOZ LEP M 07631 | SNJ | Hongping Town, Shennongjia, Hubei | Rui Cheng | China | 1515 | MK420507 | MK420698 | MK420579 | MK420874 |  | MK420740 |  |
| IOZ LEP M 07632 | SNJ | Hongping Town, Shennongjia, Hubei | Rui Cheng | China | 1515 | MK420508 |  |  |  |  |  |  |
| IOZ LEP M 07633 | SNJ | Hongping Town, Shennongjia, Hubei | Rui Cheng | China | 1515 | MK420509 | MK420699 | MK420580 | MK420875 | MK420651 | MK420741 | MK420994 |
| IOZ LEP M 07636 | WM | Liziping, Wu Mountain, Chongqing | Rui Cheng | China | 1510 | MK420510 | MK420700 | MK420581 | MK420876 | MK420652 | MK420742 | MK420995 |
| IOZ LEP M 07637 | WM | Liziping, Wu Mountain, Chongqing | Rui Cheng | China | 1510 | MK420511 |  | MK420582 | MK420877 | MK420653 | MK420743 | MK420996 |
| IOZ LEP M 07663 | HX | Hongxia Forest Farm, Ningxia | Nan Jiang | China | 1896 |  |  |  |  |  |  |  |
| IOZ LEP M 07670 | JLM | Jinggang Mountain, Jiangxi | Dayong Xue | China | 720 | MK420512 |  | MK420583 | MK420888 | MK420654 | MK420750 | MK421008 |
| IOZ LEP M 07671 | DBM | Santan, Guangshui, Hubei | Nan Jiang | China | 900 | MK420513 | MK420701 | MK420584 | MK420889 | MK420655 |  | MK421009 |
| IOZ LEP M 07797 | TMM | Sanmuping, Tianmushan, Zhejiang | Rui Cheng | China | 789 | MK420514 |  | MK420585 |  |  | MK420755 |  |
| IOZ LEP M 07798 | TMM | Tianmushan, Zhejiang | Rui Cheng | China | 789 | MK420515 |  | MK420586 |  |  | MK420756 | MK421011 |
| IOZ LEP M 07799 | TMM | Tianmushan, Zhejiang | Rui Cheng | China | 789 | MK420516 | MK420702 | MK420587 |  |  |  |  |
| IOZ LEP M 07986 | HY | Huayang, Yang County, Shaanxi | Jing Li | China | 1138 | MK420517 |  | MK420588 |  |  | MK420757 |  |
| IOZ LEP M 10630 | BJ | Baiyangcheng Village, Changping | Xiaodan Pan | China | 213 | MK420518 | MK420703 |  | MK420897 | MK420657 | MK420764 | MK421020 |
| IOZ LEP M 11626 | BYM | Baiyun Mountain, Song County, Henan | Nan Jiang | China | 815 | MK420519 | MK420704 | MK420589 | MK420898 | MK420658 | MK420765 |  |
| IOZ LEP M 12312 | JLM | Daqiutian, Jiulian Mountain, Jiangxi | Chaodong Zhu | China | 800-1430 | MK420520 |  | MK420591 |  |  | MK420767 |  |
| IOZ LEP M 12313 | JLM | Daqiutian, Jiulian Mountain, Jiangxi | Chaodong Zhu | China | 800-1430 | MK420521 |  | MK420592 |  |  |  |  |
| IOZ LEP M 12314 | JLM | Daqiutian, Jiulian Mountain, Jiangxi | Chaodong Zhu | China | 800-1430 | MK420522 |  | MK420593 |  |  |  |  |
| IOZ LEP M 12315 | JLM | Daqiutian, Jiulian Mountain, Jiangxi | Chaodong Zhu | China | 800-1430 | MK420523 |  | MK420594 |  |  |  |  |
| IOZ LEP M 12316 | JLM | Daqiutian, Jiulian Mountain, Jiangxi | Chaodong Zhu | China | 800-1430 | MK420524 |  | MK420595 |  |  |  |  |
| IOZ LEP M 12318 | JLM | Daqiutian, Jiulian Mountain, Jiangxi | Chaodong Zhu | China | 800-1430 | MK420525 |  | MK420596 |  |  |  |  |
| IOZ LEP M 12319 | JLM | Daqiutian, Jiulian Mountain, Jiangxi | Chaodong Zhu | China | 800-1430 | MK420526 |  | MK420597 |  |  |  |  |
| IOZ LEP M 12320 | JLM | Daqiutian, Jiulian Mountain, Jiangxi | Chaodong Zhu | China | 800-1430 | MK420527 | MK420705 | MK420598 |  |  |  |  |
| IOZ LEP M 12321 | JLM | Daqiutian, Jiulian Mountain,Jiangxi | Chaodong Zhu | China | 800-1430 | MK420528 |  | MK420599 |  |  |  |  |
| IOZ LEP M 12322 | JLM | Daqiutian, Jiulian Mountain, Jiangxi | Chaodong Zhu | China | 800-1430 | MK420529 | MK420706 | MK420600 |  |  |  |  |
| IOZ LEP M 12323 | JLM | Daqiutian, Jiulian Mountain, Jianxi | Chaodong Zhu | China | 800-1430 | MK420530 |  | MK420601 |  |  |  |  |
| IOZ LEP M 12325 | MH | Manghe Village, Yangcheng, Shanxi | Chaodong Zhu | China | 800-1430 | MK420531 |  |  |  |  |  |  |
| IOZ LEP M 12346 | TMM | Tianmushan, Zhejiang | Rui Cheng | China | 789 | MK420532 |  |  |  |  |  |  |
| IOZ LEP M 12712 | DBM | Taohuachong, Dabie Mountain, Hubei | Dayong Xue | China | 590 | MK420533 |  | MK420590 | MK420899 | MK420659 | MK420766 | MK421021 |
| IOZ LEP M 13384 | BJ | Xiaolongmen, Beijing | Rui Cheng | China |  |  |  | MK420602 | MK420900 | MK420660 | MK420768 | MK421022 |
| IOZ LEP M 13385 | BJ | Xiaolongmen, Beijing | Rui Cheng | China |  |  | MK420707 | MK420603 | MK420901 | MK420661 | MK420769 | MK421023 |
| IOZ LEP M 13386 | BJ | Xiaolongmen, Beijing | Rui Cheng | China |  |  |  | MK420604 | MK420902 | MK420662 | MK420770 | MK421024 |
| IOZ LEP M 18377 | HuN | Huping Mountain, Hunan | Jian Yao | China | 248-632 | MK420534 | MK420708 | MK420605 | MK420947 |  | MK420821 | MK421072 |
| IOZ LEP M 18381 | HuN | Huping Mountain, Hunan | Jian Yao | China | 248-632 | MK420535 |  | MK420606 | MK420948 |  | MK420822 | MK421073 |
| IOZ LEP M 18387 | HuN | Huping Mountain, Hunan | Jian Yao | China | 248-632 | MK420536 |  | MK420607 | MK420949 |  | MK420823 |  |
| IOZ LEP M 18396 | HuN | Huping Mountain, Hunan | Jian Yao | China | 248-632 | MK420537 |  | MK420608 | MK420950 |  | MK420824 |  |
| IOZ LEP M 18397 | HuN | Huping Mountain, Hunan | Jian Yao | China | 248-632 |  |  | MK420609 |  |  | MK420825 |  |
| IOZ LEP M 18399 | HuN | Huping Mountain, Hunan | Jian Yao | China | 248-632 | MK420538 | MK420709 | MK420610 | MK420951 |  | MK420826 |  |
| IOZ LEP M 19517 | SMM | Siming Mountain, Zhejiang | Xiaoshuang Ban | China | 853 | MK420539 |  | MK420611 |  | MK420664 | MK420827 |  |
| IOZ LEP M 19561 | SMM | Siming Mountain, Zhejiang | Rui Cheng | China | 853 | MK420540 | MK420710 | MK420612 | MK420952 | MK420665 | MK420828 |  |
| IOZ LEP M 19586 | SMM | Siming Mountain, Zhejiang | Rui Cheng | China | 853 | MK420541 | MK420711 | MK420613 | MK420953 | MK420666 | MK420829 |  |
| IOZ LEP M 21467 | DF | Defu Reserve, Guangxi | Lanbin Xiang | China | 1334 | MK420542 | MK420712 | MK420614 | MK420955 | MK420667 | MK420832 | MK421078 |
| IOZ LEP M 21797 | TC | Tengchong Wetland, Yunnan | Lanbin Xiang | China | 1730 | MK420543 | MK420713 | MK420615 | MK420956 | MK420668 | MK420834 | MK421080 |
| IOZ LEP M 21834 | TC | Tengchong Wetland, Yunnan | Xiaoshuang Ban | China | 1730 | MK420544 | MK420714 | MK420616 |  |  |  |  |
| IOZ LEP M 24920 | WDY | Wangdongyang Alpine wetland, Zhejiang | Xinxin Li | China | 1010 | MK420545 | MK420715 | MK420617 | MK420960 | MK420669 | MK420836 | MK421084 |
| IOZ LEP M 24965 | XDV | Xingdun Village, Zhejiang | Xinxin Li | China | 608 | MK420546 | MK420716 | MK420618 | MK420961 | MK420670 | MK420837 |  |
| IOZ LEP M 35252 | PQG | Pangquangou, Shanxi | Le Cui | China | 1670 | MK420547 | MK420717 | MK420619 | MK420965 | MK420671 | MK420841 |  |
| IOZ LEP M 35254 | PQG | Pangquangou, Shanxi | Shan Jiang | China | 1670 | MK420548 |  | MK420620 | MK420966 | MK420672 | MK420842 |  |
| IOZ LEP M 36711 | XJG | Xiaojinggou, Inner Mongolia | Rui Cheng | China | 1410 |  |  | MK420621 | MK420967 | MK420673 | MK420843 |  |
| IOZ LEP M 36712 | XJG | Xiaojinggou, Inner Mongolia | Rui Cheng | China | 1410 |  | MK420718 | MK420622 | MK420968 | MK420674 | MK420844 |  |
| IOZ LEP M 36930 | XJG | Xiaojinggou, Inner Mongolia | Rui Cheng | China | 1410 |  |  | MK420623 |  | MK420675 | MK420845 |  |
| IOZ LEP M 37001 | XJG | Xiaojinggou, Inner Mongolia | Shan Jiang | China | 1410 |  |  | MK420624 |  | MK420676 | MK420846 | MK421088 |
| IOZ LEP M 39233 | MM | Mianshan Mountain, Shanxi | Xinyi Zhang | China | 1359 |  |  | MK420625 |  |  | MK420847 | MK421089 |
| IOZ LEP M 39234 | MM | Mianshan Mountain, Shanxi | Xinyi Zhang | China | 1359 |  | MK420719 | MK420626 | MK420969 |  | MK420848 |  |
| IOZ LEP M 39235 | MH | Dahe Forest Farm, Shanxi | Xinyi Zhang | China | 1212 |  |  | MK420627 |  |  | MK420849 |  |
| IOZ LEP M 39236 | MH | Dahe Forest Farm, Shanxi | Xinyi Zhang | China | 1212 | MK420549 | MK420720 | MK420628 | MK420970 |  | MK420850 |  |
| IOZ LEP M 39237 | MH | Dahe Forest Farm, Shanxi | Xinyi Zhang | China | 1212 | MK420550 |  | MK420629 | MK420971 | MK420677 | MK420851 |  |
| IOZ LEP M 39238 | MH | Dahe Forest Farm. Shanxi | Xinyi Zhang | China | 1212 | MK420551 |  | MK420630 |  | MK420678 | MK420852 | MK421090 |
| IOZ LEP M 39239 | MH | Xiachuan Village, Shanxi | Xinyi Zhang | China | 1538 | MK420552 | MK420721 | MK420631 | MK420972 |  |  |  |
| IOZ LEP M 39240 | MH | Xiachuan Village, Shanxi | Xinyi Zhang | China | 1538 | MK420553 |  | MK420632 |  |  |  |  |
| IOZ LEP M 39241 | MH | Dahe Forest Farm, Shanxi | Xinyi Zhang | China | 1212 | MK420554 | MK420722 | MK420633 | MK420973 |  | MK420853 |  |
| IOZ LEP M 39242 | MH | Huangguman, Shanxi | Xinyi Zhang | China | 1258 | MK420555 | MK420723 | MK420634 | MK420974 | MK420679 | MK4208540 |  |
| IOZ LEP M 39243 | MH | Dahe Forest Farm, Shanxi | Xinyi Zhang | China | 1212 | MK420556 |  | MK420635 | MK420975 | MK420680 | MK420855 |  |
| IOZ LEP M 39244 | MH | Dahe Forest Farm, Shanxi | Xinyi Zhang | China | 1212 | MK420557 |  | MK420636 |  |  | MK420856 |  |
| IOZ LEP M 39245 | MH | Huangguman, Shanxi | Xinyi Zhang | China | 1258 | MK420558 | MK420724 | MK420637 |  |  |  |  |
| IOZ LEP M 39246 | MH | Manghe, Shanxi | Xinyi Zhang | China | 557 | MK420559 | MK420725 | MK420638 | MK420976 | MK420681 | MK420857 |  |
| IOZ LEP M 39247 | MH | Manghe, Shanxi | Xinyi Zhang | China | 557 | MK420560 |  | MK420639 | MK420977 |  | MK420858 |  |
| IOZ LEP M 39248 | MH | Manghe, Shanxi | Xinyi Zhang | China | 557 | MK420561 | MK420726 | MK420640 |  | MK420682 | MK420859 |  |
| IOZ LEP M 39249 | MH | Manghe, Shanxi | Xinyi Zhang | China | 557 | MK420562 |  | MK420641 | MK420978 | MK420683 | MK420860 |  |
| IOZ LEP M 39250 | MH | Manghe, Shanxi | Xinyi Zhang | China | 557 | MK420563 |  | MK420642 | MK420979 | MK420684 | MK420861 |  |
| IOZ LEP M 40316 | CK | Chengkou County, Chongqing | Rui Cheng | China |  | MK420564 |  |  |  |  |  |  |

Table S2 Total primers used in this study.

| Region | Primer pairs (F/R) | Sequence (forward and reverse) 5' → 3' | Source |
| --- | --- | --- | --- |
| COI | LCO1490 | GGTCAACAAATCATAAAGATATTGG | Folmer et al., 1994 |
|  | HCO2198 | TAAACTTCAGGGTGACCAAAAAATCA | Folmer et al., 1994 |
| CYTB | CP1 | GATGATGAAATTTTGGATC | Sezonlin et al., 2006 |
|  | TRS | TATTTCTTTATTATGTTTTCAAAAC | Simon et al., 1994 |
| ND5 | OurND5-F | TAAATAGCCCCATTAAAACGACATA | This study |
|  | OurND5-R | ACCAGTCTCAGCTTTAGTACATTCTT | This study |
| COII | Patrick | CTAATATGGCAGATTATATGTAATGGA | Caterino & Sperling, 1999 |
|  | Eva | GAGACCATTACTTGCTTTCGATCATCT | Caterino & Sperling, 1999 |
| EF-1a | EF1alepF2 | ACAAATGCGGTGGTATCGACAA | Yamamoto & Sota, 2007 |
|  | EF1aR | GATTTACCRGWACGACGRTC | Kawakita et al., 2004 |
| GAPDH | GAPDH-F3 | CAAGGCTGGTGCTGAATACGTC | Li et al., 2017 |
|  | GAPDH-R3 | CTTGGTCTGGATGTACTTGATGAGAT | Li et al., 2017 |
| CAD | CAD150519F | YATAGTYGTWGCACCTAGTCAAAC | Jiang et al., 2017 |
|  | CAD150519R2 | ARTAAAGMCKATCRCTCATATCGTAGTC | Jiang et al., 2017 |

Reference

Caterino, M. S., & Sperling, F. A. (1999). *Papilio* Phylogeny based on mitochondrial cytochrome oxidase I and II genes. *Molecular Phylogenetics and Evolution*, *11*, 122–137.

Folmer, O., Black, M., Hoeh, W., Lutz, R., & Vrijenhoek, R. (1994). DNA primers for amplification of mitochondrial cytochrome c oxidase subunit I from diverse metazoan invertebrates. *Molecular Marine Biology and Biotechnology*, 3, 294–299.

Jiang, N., Li, X., Hausmann, A., Cheng, R., Xue, D., & Han, H. (2017). A molecular phylogeny of the Palaearctic and Oriental members of the tribe Boarmiini (Lepidoptera: Geometridae: Ennominae). *Invertebrate Systematics*, *31*, 427-441.

Kawakita, A., Takimura, A., Terachi, T., Sota, T., & Kato, M. (2004). Cospeciation analysis of an obligate pollination mutualism: have Glochidion trees (Euphorbiaceae) and pollinating *Epicephala* moths (Gracillariidae) diversified in parallel? *Evolution*, *58*, 2201–2214.

Li, X., Jiang, N., Cheng, R., Xue, D., Qu, Y., & Han, H. (2017). Allopatric divergence and secondary contact without genetic admixture for *Arichanna perimelaina* (Lepidoptera: Geometridae), an alpine moth endemic to the Hengduan Mountains. *Systematic Entomology*, *42*, 703–713.

Sezonlin, M., Dupas, S., Le Rü, B., Le Gall, P., Moyal, P., Calatayud, P. A., Giffard, I., & Silvain, J. F. (2006). Phylogeography and population genetics of the maize stalk borer Busseola fusca (Lepidoptera, Noctuidae) in sub-Saharan Africa. *Molecular Ecology*, *15*, 407–420.

Simon, C., Frati, F., Beckenbach, A., Crespi, B., Liu, H., & Flook, P. (1994). Evolution, weighting and phylogenetic utility of mitochondrial gene sequences and a compilation of conserved polymerase chain reaction primers. *Annals of the Entomological Society of America*, *87*, 651–701.

Yamamoto, S., & Sota, T. (2007). Phylogeny of the Geometridae and the evolution of winter moths inferred from a simultaneous analysis of mitochondrial and nuclear genes. *Molecular Phylogenetics and Evolution*, *44*, 711–723.

Figure S1 The phylogenetic tree of *Ourapteryx szechuana* based on three single nuDNA gene. Colors are congruent with different lineages.


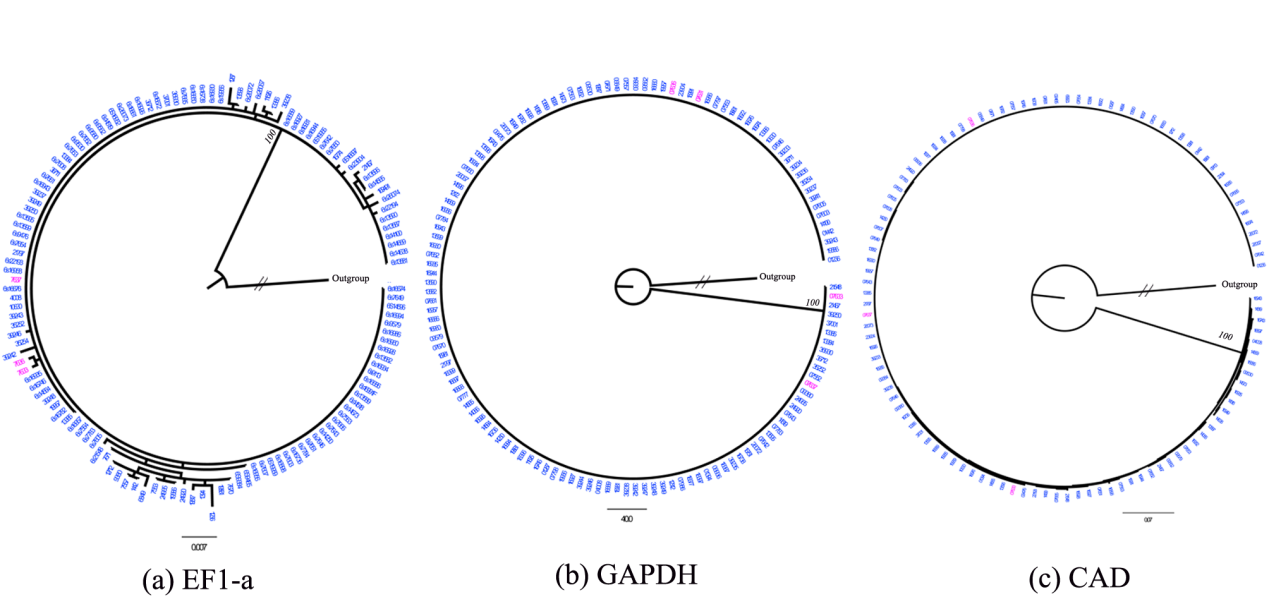

Supplement: Supplementary file 1 — Supplementary Material [file ECE3-11-10066-s001.docx]
